# Toll-like receptor 9 and the inflammatory response to surgical trauma and cardiopulmonary bypass

**DOI:** 10.1186/s13019-020-01179-y

**Published:** 2020-06-11

**Authors:** Hatam Naase, Leanne Harling, Emaddin Kidher, Amir Sepehripour, Bao Nguyen, Alkistis Kapelouzou, Dennis Cokkinos, George Stavridis, Gianni Angelini, Paul C. Evans, Thanos Athanasiou

**Affiliations:** 1 Department of Surgery and Cancer, Imperial College London, St Mary’s Hospital, London, W2 1NY UK; 2 Imperial College Healthcare NHS Trust, Hammersmith Hospital, London, UK; 3 National Heart and Lung Institute, Imperial College London, London, UK; 4 Biomedical Research Foundation, Academy of Athens, Athens, Greece; 5 Department of Cardiovascular Science, University of Sheffield, Sheffield, UK

**Keywords:** Toll-like receptors, TLR9, Surgical trauma, Cardiopulmonary bypass, Cardiac surgery, Inflammatory response

## Abstract

**Objectives:**

Cardiac surgery can lead to post-operative end-organ complications secondary to activation of systemic inflammatory response. We hypothesize that surgical trauma or cardiopulmonary bypass (CPB) may initiate systemic inflammatory response via release of mitochondrial DNA (mtDNA) signaling Toll-like receptor 9 (TLR9) and interleukin-6 production (IL-6).

**Materials and methods:**

The role of TLR9 in systemic inflammatory response in cardiac surgery was studied using a murine model of sternotomy and a porcine model of sternotomy and CPB. mtDNA and IL-6 were measured with and without TLR9-antagonist treatment. To study ischemia-reperfusion injury, we utilized an ex-vivo porcine kidney model.

**Results:**

In the rodent model (*n* = 15), circulating mtDNA increased 19-fold (19.29 ± 3.31, *p* < 0.001) and plasma IL-6 levels increased 59-fold (59.06 ± 14.98) at 1-min post-sternotomy compared to pre-sternotomy. In the murine model (*n* = 11), administration of TLR-9 antagonists lowered IL-6 expression post-sternotomy when compared to controls (59.06 ± 14.98 vs. 5.25 ± 1.08) indicating that TLR-9 is a positive regulator of IL-6 after sternotomy. Using porcine models (*n* = 10), a significant increase in circulating mtDNA was observed after CPB (Fold change 29.9 ± 4.8, *p* = 0.005) and along with IL-6 following renal ischaemia-reperfusion. Addition of the antioxidant sulforaphane reduced circulating mtDNA when compared to controls (FC 7.36 ± 0.61 vs. 32.0 ± 4.17 at 60 min post-CPB).

**Conclusion:**

CPB, surgical trauma and ischemic perfusion injury trigger the release of circulating mtDNA that activates TLR-9, in turn stimulating a release of IL-6. Therefore, TLR-9 antagonists may attenuate this response and may provide a future therapeutic target whereby the systemic inflammatory response to cardiac surgery may be manipulated to improve clinical outcomes.

## Introduction

Cardiac surgery with the use of cardiopulmonary bypass (CPB) has been associated with a systemic inflammatory response potentially leading to significant morbidity and mortality. This response is multi-factorial involving the exposure of circulating blood to artificial surfaces of the extracorporeal circuit, non-physiological shear stress of the CPB roller pump, hypothermia, and a potentially inevitable ischemia-reperfusion injury (IRI) [1, 2]. The subsequent activation of a series of inflammatory cascades [3] can be both further initiated and exacerbated by significant tissue injury incurred during CPB, and worsened by the release of endogenous inflammatory mediators [4]. The clinical sequelae of such a pro-inflammatory state result in a hyper-dynamic circulation, increase in the cardiac output and decrease in the systemic vascular resistance, prompting the need for inotropic and vasoconstrictive support [5]. Despite several significant improvements in the CPB circuit such as heparin coated circuit, the complications secondary to tissue damage and inflammatory response still remain, and can have a profound impact on post-operative outcomes [6].

Toll like receptors are a superfamily of pattern recognition receptors, a class of proteins acting as first-line in the defense against pathogens. These proteins act to recognize both pathogen-associated and damage-associated molecular patterns (DAMPs), playing a significant role in inflammation, cell immune regulation and proliferation [7, 8]. Sources of DAMPs in sterile inflammation can be found in the mitochondrion, specifically the mitochondrial DNA (mtDNA), which also contains CpG-DNA repeats [9, 10]. During mechanical injury or ischemia, necrosis and/or cellular apoptosis ultimately results in mitochondrial damage and leak of mitochondrial contents into the bloodstream [9, 11, 12]. This leads to the potential signaling of TLR9, which recognizes unmethylated CpG sequences in DNA molecules [13]. As such, TLR signaling provides a potential pathway by which the inflammatory response to CPB may be mediated, and a better understanding of this relationship may in future enable us to provide a translational benefit in clinical outcomes.

Primary cardiac surgical trauma, CPB and ischaemia reperfusion injury (IRI) may all initiate the systemic inflammatory response. In this manuscript we aim to systematically address these mechanisms by examining the release of mtDNA and determining TLR9 as an effector of IL-6 expression. Finally, we evaluate the role of pretreatment with the anti-inflammatory and anti-oxidant substance sulforaphane, as a means of dampening the inflammatory response in vitro with view to future translation into in vivo studies and subsequent clinical practice.

## Material & Methods

### In vitro model

#### Purification of total mtDNA

In all animal models, DNA Purification from Blood or Body Fluids was performed using the QIAamp DNA Blood Mini Kit (Qiagen®) according to the manufacturer protocol [14]. In summary, DNA was extracted from 200 μl of plasma and concentrated by double elution with 50 μl AE buffer. To detect DNA purity for any protein contamination, the A_260/280_ ratio of the DNA was assessed using the NanoDrop® spectrophotometer, which was between 1.7–1.9 excluding any contamination.

Quantitative real-time polymerase chain reaction (RT-qPCR) was used to measure mtDNA levels using primers that recognized the mitochondrial gene cytochrome C oxidase subunit III in rat, mouse or pig (Table 1). The reaction was prepared using 1 μl of DNA in each tested well plus 12.5 μl of SYBR Green PCR Master Mix (Taqman, Applied Biosystems, Life Technologies, Paisley, UK), 0.5 μl of mtDNA primer and 10.5 μl of water. For each study probe identical RT-qPCR reactions were also carried out using a sterile water negative control and a HUVEC mtDNA positive control.

**Table 1:** Quantitative real-time polymerase chain reaction (RT-qPCR) primers used in small and large animal models

Gene Specific Primer	Forward	Reverse
***Mouse***		
Interleukin-6	CCTCTGGTCTTCTGGAGTACC	ACTCCTTCTGTGACTCCAGC
Cytochrome oxidase C	AAAGTTGAACTGTACCCCTTAAGT	CACTCATTTGTGCTCCTGTTCATC
***Rodent***		
Cytochrome oxidase C	ACATACCAAGGCCACCAAC	CAGAAAAATCCGGCAAAGAA
***Pig***		
Cytochrome oxidase C	CCCATTATGATTGGGGGTTT	TGCTGTGTATGCGTCAGGAT

All reactions were carried out in three technical replicates using the Applied Biosystems 7500 fast Real-Time PCR System [15]. Amplification plots were examined for adequate amplification and successful PCR reaction.

### Animal models

All animals received humane care in compliance with the Principles of Laboratory Animal Care and according to UK Home Office regulations and approved by local ethical Central Ethical Review Process Committee known as Animal Welfare and Ethical Review Body (AWERB).

#### Rat model

Adult male Sprague–Dawley rats (*n* = 15) (400-450 g) were acclimatized prior to study and housed in individually ventilated cages with no more than 3 per cage for 2 weeks. During the procedure the rats were anaesthetized using intraperitoneal ketamine (50 mg/kg) and xylazin (2 mg/kg), tracheostomy and endotracheal intubation with 14G cannula and mechanical ventilation for 15–20 min. At the end of the experiment, the animals were euthanized using intravenous barbiturate overdose. To detect the circulating level of the mtDNA through mimicking the cardiac surgical access, midline sternotomy was performed where initially the skin was shaved then 2 cm midline skin incision above the sternal was performed and gentle dissection of subcutaneous tissue and lifting the sternum with forceps and using scissors to perform the sternotomy, care was taken to preserve the pleura to avoid pneumothorax. Tail vein blood samples were collected separately at pre-sternotomy and 1- and 10-min post-sternotomy.

#### Mouse model- response to TLR9 suppression with ODN2088

Eleven male C57BL/6, 9–10-week-old mice (6 control; 5 experimental) were acclimatized prior to the study and housed in individually ventilated cages with no more than 4 mice per cage for 2 weeks. Test mice were injected with intra-peritoneal mouse-preferred synthetic TLR-9 antagonist oligodeoxynucleotide ODN2088 (InvivoGene®, San Diego, CA, USA) (100 μg/25 g body weight) prior to induction of anesthesia. An equal volume of 0.9% saline was used for the control group. Mice were anaesthetized using ketamine (50 mg/kg) and xylazin (2 mg/kg) and intubation was done through neck incision. Shaving skin then 1 cm midline skin incision above the sternal was performed and gentle dissection of subcutaneous tissue and lifting the sternum with forceps and using scissors to perform the mid line sternotomy, care was taken to preserve the pleura to avoid pneumothorax. Two pre-sternotomy blood samples were collected, before and after intubation. A third sample was taken immediately after sternotomy. At the end of the experiment, euthanasia was done by cervical dislocation.

#### Large animal porcine model

In the large animal setting we proceeded with performing CPB with the same bypass circuit systems that are used in humans using Stöckert multiflow roller pump (Sorin Group GmbH, Munich, Germany). Surgical approach was through midline sternotomy using a Gigli saw, standard systemic heparinization was performed (300 IU/kg) achieving activated clotting time (ACT) greater than 400 s. CPB arterial cannulation was performed through ascending aortic when ACT> 400 s. The venous drainage was through right atrial cannulation using two-stage venous cannula, directing the distal end towards the inferior vena cava. Non-pulsatile CPB was subsequently maintained under normal pigs’ temperature (38-39C°) for 2 h with flow of 2 to 4 L/min and line pressure < 300 mmHg with disconnected lung ventilation.

In the first part of the experiment was to test the effect of circulating mtDNA in pigs in relation to CPB. Total of ten pigs underwent median sternotomy (represented by pre-CPB time point), half had CPB (*n* = 5) and half sham mechanically ventilated pigs underwent median sternotomy alone (*n* = 5). Five ml blood sampling was taken at different time-points in relation to CPB commencement (pre, end of CPB, and 90-min after CPB). Second part of the experiment includes total of ten pigs underwent CPB, half were pre-treated with 2 mg/kg antioxidants sulforaphane (*n* = 5) versus normal saline (*n* = 5). Five ml blood sampling was performed at different time-points in relation to CPB commencement (pre, at onset of CPB, at 60-min and 120-min of CPB). Plasma sample was separated by centrifugation of blood (10 min at 3000 x g) and stored at − 80 °C until further processing. The mtDNA was extracted and RT-qPCR was performed as outlined above.

#### Antioxidant response

Ten female Landrace pigs (50–60 kg) were used according to UK Home Office regulations and Directive 2010/63/EU of the European Parliament and in compliance with the Guide for the Care and Use of Laboratory Animals. Ketamine (20 mg/kg)/xyalzine (2 mg/kg) was used for sedation prior to induction of anesthesia with 5% isoflurane, followed by standard endotracheal intubation. Five pigs were then treated immediately with IV injection of Saline and the other 5 with IV sulforaphane (2 mg/kg) (the results in relation to sulforaphane in this study were published by Nguyen et al. where the author investigated the phosphorylation of p38 MAPK and NF-kB in relation to antioxidant sulforaphane pre-treatment) [16]. mtDNA was extracted from plasma as per the previous experiment; RT-PCR was performed using each samples in triplicate as described in *2.1.1*.

### Ischemia-reperfusion injury (IRI)

IRI was assessed in an ex-vivo renal reperfusion model. Autologous whole blood and perfusate were used to simulate the cold ischemia prior to implantation, followed by warm reperfusion. Where Warm ischemic injury is sustained in the time between donor cardiac arrest and organ removal. This is followed by further cold ischemic damage that ensues after flushing and storage of the allograft with cold preservation solution, and then transport to the recipient center. This simulation closely resembles the ischemia and reperfusion when the aorta is cross-clamped and then released during CPB.

Thirteen porcine kidneys were retrieved from cadaveric pigs at an abattoir, flushed with cold storage University of Wisconsin (UW) solution and placed on ice for transport back to the laboratory. Kidneys were then perfused on a modified Waters Medical (RM3) perfusion machine at 4 °C with UW solution for 6-h and then underwent autologous whole blood normothermic perfusion for 6-h. Perfusate samples during hypothermic and normothermic perfusion will be collected for determination of damage biomarkers. DNA was extracted from plasma at different time-points of reperfusion (pre, 5-min, 20-min, 2-h, 4-h and 6-h). RT-PCR with cytochrome C mitochondrial primer was employed to detect the level of circulating mtDNA. IL-6 was measured as previously described.

### Statistical analysis

Statistical analysis was conducted using GraphPad Prism version 8.1.2. Results are expressed as means ± SE at each time point for each group, *P*-values < 0.050 were considered statistically significant. Due to the nature of the experiments with small number of animals, non-parametric comparative analysis was used. Wilcoxon signed rank tests were used for matched pairs and, where relevant, p values were adjusted for multiple-comparison using Bonferroni correction methodology. For comparisons with more than 2 groups, Friedman test was used with correction for multiple comparison employed using Dunn’s methodology.

## Results

### Sternotomy enhanced circulating mtDNA in a rodent model

**Fig. 1:**
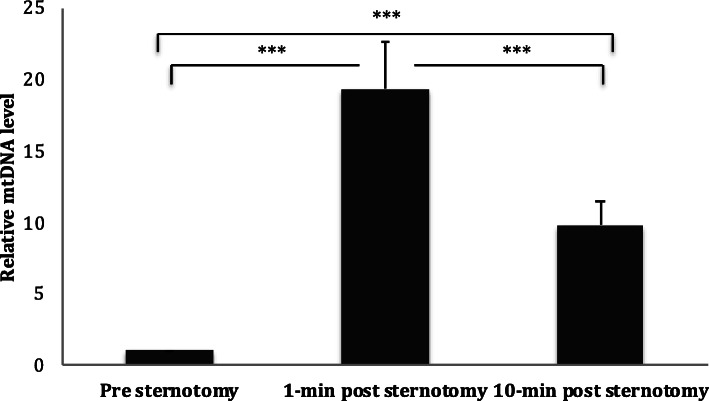
Circulating mtDNA level in response to sternotomy in the rodent model. Plasma level of circulating mtDNA in the rodent model undergoing median sternotomy at different time points. Circulating mtDNA levels peaked immediately after sternotomy

Circulating mtDNA demonstrated a significant 19-fold increase immediately post sternotomy (At 1-min: FC 19.29 ± 3.31; *p* < 0.001). At 10 min post-sternotomy, mtDNA had fallen to FC 9.79 ± 1.61; but remained statistically significant when compared to control (*p* < 0.019), suggesting a significant response of plasma mtDNA to surgical trauma (Fig. 1).

### TLR9 antagonism reduced circulating IL-6 levels in response to surgical induction in a murine model

**Fig. 2:**
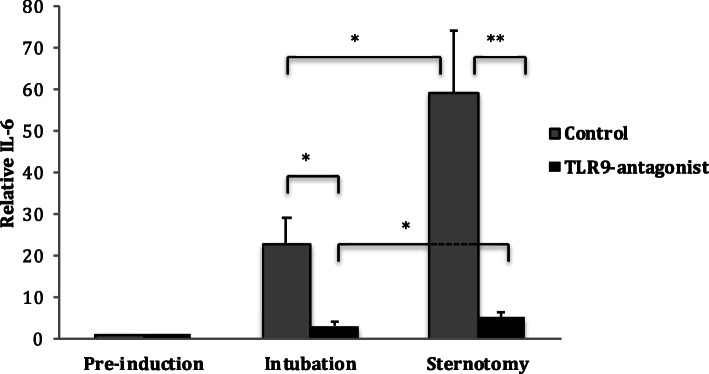
Level of circulating IL-6 in response to sternotomy in the murine model. Plasma level of IL-6 in the murine model undergoing median sternotomy at different time-points with and without pre-treatment with TLR9-antagonist (ODN2088). The plasma level of IL-6 significantly decreased when TLR9 blocked by OND2088

Anesthesia and intubation resulted in an increase in IL-6 expression when compared to pre-induction levels in the control (FC 25.8 ± 6.13) and the anti-TLR9 (FC 3.22 ± 0.68) animals; however, this increase was much lower in the TLR9-antagonist group (Fig. 2).

A further increase in IL-6 expression was seen in both groups after sternotomy, however this rise was again dampened in the TLR9-antagonist group (FC 5.25 ± 1) compared to control group (FC 59.06 ± 14.99) (Fig. 2). These data indicate that TLR9 is a positive regulator of IL-6 production in response to surgical trauma.

### Sulforaphane reduced mtDNA release in response to surgical trauma

**Fig. 3:**
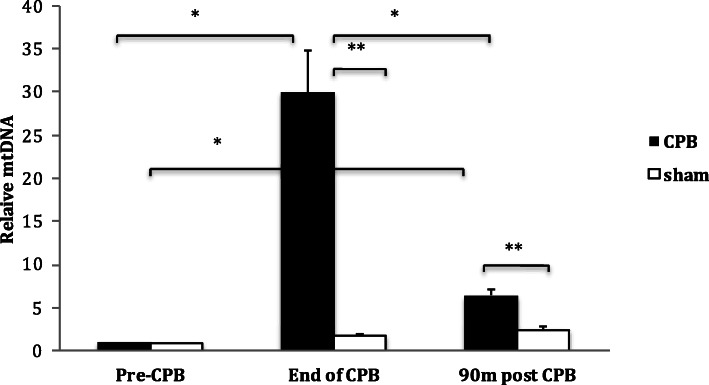
Circulating mtDNA level in response to CPB in the porcine model. Plasma level of circulating mtDNA in the porcine model undergoing median sternotomy with CPB at different time-points vs. sham

In the porcine model, circulating mtDNA increased at the end of CPB (FC 29.9 ± 4.79; *p* = 0.005) when compared to pre-operative levels, however this rise was transient and had reduced by 90-min post-CPB when compared to end of CPB levels (FC 6.47 ± 0.57, *p* = 0.34) (Fig. 3). The sham group showed a minimal increase in circulating mtDNA at the end of CPB time-point equivalent (FC 1.68 ± 0.23, *P* = 0.17), followed by a further increase at the 90-min post-CPB time-point equivalent (2.41 ± 0.42, *p* = 0.013) compared to baseline (Fig. 3).

**Fig. 4:**
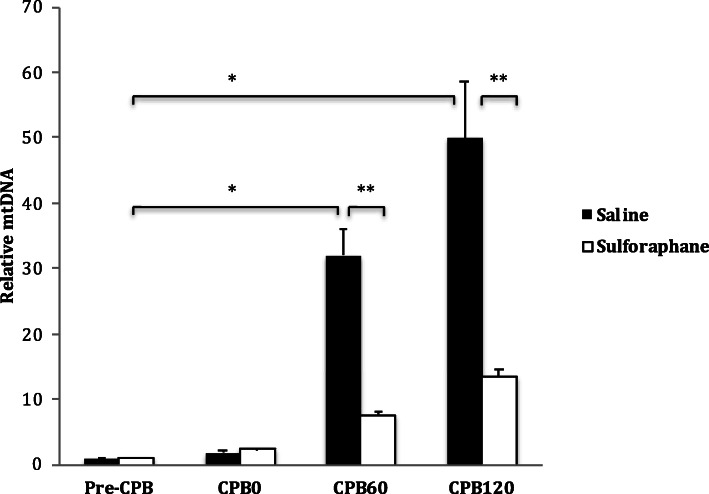
Effect of sulforaphane on mtDNA levels in CPB in the porcine model. Plasma levels of circulating mtDNA in the porcine CPB model at different time-points with or without sulforaphane pre-treatment. There was significant reduction in mtDNA levels in response to sulforaphane pre-treatment

Both sulforaphane and non-sulforaphane groups showed increases in circulating mtDNA levels at all timepoints compared to pre-CPB levels. However, sulforaphane significantly reduced the concentration of circulating mtDNA at both 60- (FC 7.36 ± 0.61 vs. 32.0 ± 4.17) and 120- (FC 13.4 ± 1.2 vs. 49.9 ± 8.6) minutes after CPB when compared to the non-sulforaphane group (Fig. 4).

### Ischemia-reperfusion injury

**Fig. 5:**
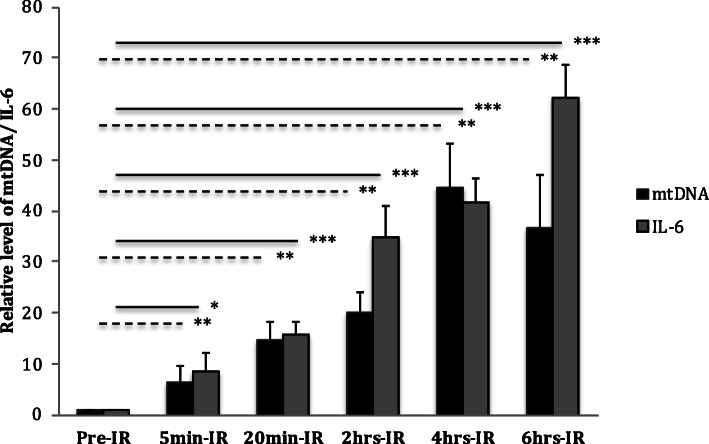
Effect of ischemic-perfusion on mtDNA nad IL-6 in porcine kidneys. Plasma level of circulating mtDNA and IL-6 in ex-vivo porcine kidney model at different time points of IRI (Dotted lines represent mtDNA and solid lines represent IL-6). All data shown are mean ± SE (* = *p <* 0.05, ** = *p <* 0.001, ***p < 0.0001)

Circulating mtDNA showed a steady increase up to 4 h after reperfusion in kidneys that were perfused ex vivo. IL-6 also increased during reperfusion throughout the study period. At 6 h after reperfusion, circulating mtDNA levels began to decline whilst IL-6 remained significantly elevated when compared to pre-perfusion levels (Fig. 5).

## Discussion

Cardiopulmonary bypass, surgical trauma and the associated ischemia-reperfusion injury (IRI) may all activate the inflammatory response to cardiac surgery, potentially causing significant morbidity [1, 17–20]. However, the mechanisms involved are still not completely understood. Mitochondrial damage associated proteins (DAMPs) have been demonstrated as inflammatory modulators leading to tissue damage in a variety of pathological conditions including systemic inflammatory response syndrome (SIRS), connective tissue diseases, myocardial infarction, vascular dysfunction and in individuals receiving chemotherapy and hemodialysis [21–23].

In this study we addressed the role of surgery-induced mitochondrial damage in the downstream signaling of the pro-inflammatory cytokine IL-6 via the TLR-9 pathway. We have confirmed the accumulation of mitochondrial DNA in the circulation using several in vivo post-sternotomy/cardiopulmonary bypass models. Furthermore, we have shown that blockade of TLR-9 in vivo results in a significant decrease in IL-6 expression thus revealing a direct relationship between TLR-9 signaling subsequent IL-6 driven inflammatory pathways in surgical trauma.

Post-cardiac surgery, inflammation is thought to be related to a triad of surgical stimuli including CPB, surgical trauma and ischaemia-reperfusion. Addressing each point in turn we demonstrate mitochondrial damage and subsequent mtDNA release in all three components of this triad. However, our results suggest these factors are not equally weighted in driving inflammation, with mtDNA release far more strongly related to CPB than surgical trauma in our large animal model. Furthermore, longer durations of CPB continued to drive mitochondrial damage, leading to an ongoing increase in mtDNA release suggesting a correlation between overall bypass time and the post-operative inflammatory response. Some studies showed cardiac surgery with long-CPB (> 100 min) has higher mtDNA levels than short-CPB (< 100 min) [24].

It has been demonstrated that mtDNA activates neutrophils throughTLR9/p38 MAPK [10] and enhance TLR9 expression in lung tissue in rats [25]. Our study also confirms the relationship between mitochondrial damage, as measured by circulating mtDNA release, and the signaling of TLR9 in a controlled animal model. TLR9 recognizes bacterial DNA motifs [13] and as mtDNA has a similar molecular signature to bacterial DNA this subsequently signals downstream TLR9 mediated inflammatory pathways [26]. Recent studies have now shown this remains true in humans, with an average 6-fold increase in circulating mtDNA observed following CPB when compared to pre-CPB levels in one study [27]. Similarly, this work demonstrated an increase in IL-6 levels following CPB which continued to rise to reach significance 1–2 days after surgery suggesting rises in circulating mtDNA may act as a means of predicting the future inflammatory response and its associated complications [27].

In order to examine the final component of the triad of surgical drivers of inflammation we examined ischaemia-reperfusion in an ex-vivo porcine kidney model. This translates to an important driver of postoperative morbidity in clinical practice, particularly in patients undergoing cardiac surgery with cardiopulmonary bypass and cardioplegic arrest [18]. Our results demonstrate that renal IRI leads to an increase in mtDNA and the pro-inflammatory cytokine IL-6. IL-6 may in turn activate intracellular signaling and stimulate leukocyte-endothelial interaction leading to leukocyte migration and tissue damage [28].

### Translating mechanism into future targeted anti-inflammatory therapies

We were able to successfully attenuate TLR9 signaling through the addition of selective TLR9 antagonists ODN-2088 in the murine model, leading to a significant reduction of circulating IL-6 gene expression in vivo. As such, direct manipulation of this pathway is of potential therapeutic benefit when considering attenuation of the inflammatory response. Currently, a Phase I clinical trial is underway utilizing the TLR-7, − 8 and − 9 antagonist IMO-8400 to reduce systemic inflammatory drivers in autoimmune disease [29]. These results will be eagerly awaited, with the potential for this or similar therapies to have a role in the treatment for selected cases of post cardiac surgery SIRS.

It has previously been demonstrated by co-authors that the antioxidant sulforaphane acts to reduce pro-inflammatory p38 and nuclear factor-κB activation, suppressing inflammatory cytokine expression in a porcine CPB model [16]. This study demonstrates that pre-treatment with sulforaphane may also act through reducing circulating mtDNA release. Although further work is necessary to address this mechanism in more detail, it is likely this occurs at least in part secondary to a reduction in cellular apoptosis combined with intracellular protection against mitochondrial depolarization and DNA fragmentation [30]. These results suggest a potential therapeutic role for treatment with sulforaphane in the reduction of the systemic inflammatory response to all components of the triad of cardiac surgical stimuli.

### Limitations

A key limitation of this study is that we were not able to assess the role of TLR9 antagonization in an in vivo combined CPB-IRI model. Such a model would be most analogous to human cardiac surgery and therefore requires further exploration in subsequent work. Bigger sample size experiment with additional parameter of systemic inflammatory reaction such as cardioplegia to achieve myocardial arrest would be beneficial. Although outside the scope of this study, other interesting considerations would be to determine the impact of alternative operative strategies such as off pump cardiac surgery on pro-inflammatory signaling when compared to conventional CPB. Furthermore, future translational work may build on the role of anti-TLR9 targeted anti-inflammatory therapies in Phase I clinical trials, with the potential to ultimately reduce the downstream clinical complications of the post-surgical systemic pro-inflammatory state.

## Conclusions

This study demonstrates a direct link between all components of the triad of cardiac surgical stressors and mitochondrial damage. Subsequent release of circulating mtDNA activates and signals TLR9, which in turn directly stimulates a release of IL-6, leading to endothelial activation and leukocyte migration. Manipulation of this pathway by means of a TLR9 antagonist was able to attenuate this response both in vitro and in vivo*,* and may provide a future therapeutic target whereby the systemic inflammatory response to cardiac surgery may be manipulated to improve clinical outcomes.

## Data Availability

The dataset used and/or analysed during the current study are available from corresponding author on reasonable request.

## Abbreviations

ACT: Activated clotting time
AWERB: Animal Welfare and Ethical Review Body
CPB: Cardiopulmonary bypass
DAMP: Damage-associated molecular patterns
FC: Fold-change
IRI: Ischemic reperfusion injury
IL-6: Interleukin-6
MAPK: Mitogen-activated protein kinases
mtDNA: Mitochondrial DNA
NF-κB: Nuclear factor kappa-light-chain-enhancer of activated B cells
RT-qPCR: Real time quantitative polymerase chain reaction
SIRS: Systemic inflammatory response syndrom
TLR9: Toll-like receptor 9
